# ‘Fend for yourself’: Nurses’ experiences transitioning to Family Care Team practice settings in Newfoundland and Labrador

**DOI:** 10.1371/journal.pone.0345818

**Published:** 2026-03-24

**Authors:** Robin Devey-Burry, Julia Lukewich, Dana Ryan, Myuri Sivanthan, Maria Mathews, Marie-Eve Poitras, Cheryl Etchegary, Shabnam Asghari, Margot Antle

**Affiliations:** 1 Faculty of Nursing, Memorial University of Newfoundland, St. John’s, Newfoundland and Labrador, Canada; 2 Department of Family Medicine, Schulich School of Medicine & Dentistry, Western University, London, Ontario, Canada; 3 Département de Médecine de Famille et Médecine d’Urgence, Faculté de Médecine et des Sciences de la Santé, Université de Sherbrooke, Sherbrooke, Québec, Canada; 4 Quality of Care NL, Faculty of Medicine, Memorial University of Newfoundland, St. John’s, Newfoundland and Labrador, Canada; 5 Discipline of Family Medicine, Faculty of Medicine, Memorial University of Newfoundland, St. John’s, Newfoundland and Labrador, Canada; 6 Newfoundland and Labrador Nurse Practitioner Association, St. John’s, Newfoundland and Labrador, Canada; University of New Brunswick, CANADA

## Abstract

**Background:**

Family Care Teams were introduced in Newfoundland and Labrador (NL) as a strategy to strengthen primary care through team-based models that optimize interprofessional collaboration. Nurses, including nurse practitioners (NPs), registered nurses (RNs), and licensed practical nurses (LPNs), play critical roles in these models; however, little is known about nurses’ transition to these settings or the supports shaping their integration and effectiveness. To address this gap, we explored nurses’ experiences transitioning into Family Care Teams, including supports for integration and the barriers and facilitators influencing this process.

**Methods:**

As part of a qualitative descriptive study, we conducted semi-structured interviews with 25 nurses (6 NPs, 13 RNs, 6 LPNs) employed in Family Care Teams across five NL health zones. During the interviews, nurses described their experiences working in Family Care Teams, available practice supports, current roles, and barriers and facilitators to maximizing scope of practice. Interviews were transcribed and analyzed using qualitative content analysis and constant comparison.

**Results:**

Participants described their transition to Family Care Teams in two stages: 1) orientation and 2) supportive learning relationships. Orientation was highly variable, ranging in length and structure. Learning in this area was often self-directed, technology-focused, and asynchronous, with limited emphasis on clinical preparation or role expectations. Mentorship and shadowing opportunities were inconsistently available, with many nurses lacking access to experienced role models within the newly established teams. These gaps contributed to role ambiguity, underutilization of nursing scope of practice, and prolonged adjustment periods.

**Conclusions:**

Our findings reveal gaps in orientation and mentorship during nurses’ transition into Family Care Teams in NL. A common yet adaptable transition framework, expanded student placements, and structured mentorship are critical to optimizing nursing roles in team-based care. Strengthening practice supports and clarifying nursing contributions can improve access and care quality while informing broader initiatives to support nurses’ transition into primary care.

## Introduction

### Background

Primary care is the first point of access to comprehensive, patient-centered care that emphasizes health promotion, disease prevention, and the diagnosis and treatment of illness and injury, typically delivered by physicians and nurses in a coordinated relationship with individuals and families across the lifespan [1–5]. An effective, high-quality primary care system has the potential to offer many benefits to Canadians and to healthcare systems as a whole. Despite these benefits, the province of Newfoundland and Labrador (NL) is currently facing many challenges with respect to primary care, including limited access to services, a reliance on fee-for-service physician funding models, a high burden of chronic disease, and frequent turnover of family physicians, especially in rural/remote regions. These challenges have resulted in a lack of services and support for many patients across the province.

Team-based primary care, defined as “multiple health workers from different professional backgrounds working together with patients, families, caregivers, and communities to deliver the highest quality of care”, serves as a promising strategy to address the complex healthcare needs of patients in NL, across Canada, and globally [6]. Team-based care must involve coordinated collaboration of healthcare providers, such as family physicians, nurse practitioners (NPs), registered nurses (RNs), licensed practical nurses (LPNs), pharmacists, and social workers, to deliver patient-centered care. When delivered optimally, this team-based primary care strategy has been linked to greatly improved health outcomes, patient satisfaction, patient access to primary care services, and healthcare costs [7].

In NL, the implementation of Family Care Teams has been identified as a key strategy to strengthen primary care and address existing system gaps. Since their launch in 2022, 21 Family Care Teams have been established across the province, with plans underway for the implementation of several more [8,9]. As Family Care Teams continue to expand, many team leads, program managers, and clinical nurses have expressed a need for resources and supports to inform the integration and optimization of the nursing role within these team-based models.

### Literature review

In Canada, there are three regulated designations of nurses that typically work in a primary care setting and carry out team-based care. NPs are masters-prepared and deliver care autonomously. NPs can prescribe medications, order laboratory tests, diagnose, and perform a wide range of clinical duties beyond the scope of practice of an RN [10]. RNs are baccalaureate-prepared and work both autonomously and in collaboration with other providers to coordinate care and deliver direct health care services to patients [11]. LPNs are diploma-prepared and provide routine care for patients in predictable conditions and have a narrower scope of practice than that of NPs and RNs [12]. Nurses in primary care work in partnership with family physicians and other healthcare providers, and provide a broad range of health services, including but not limited to triage; prenatal, well-baby, and well-women care; routine immunizations; mental health and addictions support; treatment of acute illness; sexual health care; chronic disease prevention and management; health education and self-management support; coordination and implementation of targeted primary care programs; and patient navigation [13–15]. Emerging evidence shows high-performing primary care teams that include nurses are able to overcome pressures placed upon our healthcare systems by improving access and continuity of care, increasing patient outcomes and satisfaction, and improving cost effectiveness [7,16–24].

In many Canadian provinces, nurses form the core of primary care teams [16,25–28]; however, in NL, the widespread implementation of team-based care remains limited and the integration of nurses has progressed more slowly than in other parts of the country. There is scant evidence related to the experiences of nurses transitioning to team-based primary care practice, particularly with respect to the integration of RNs and LPNs within the newly established Family Care Teams in NL. It has been reported that RNs in Family Care Teams are being underutilized, which reflects findings from other regions where suboptimal integration and limited use of nurses’ full scope of practice in primary care have also been documented [29,30]. Furthermore, while efforts to integrate nurses of all designations into primary care settings in NL are increasing, the preparation and transition supports available to them remain limited, are not uniformly implemented, and have limited potential to maximize their scope of practice. Additionally, LPNs are notably underrepresented in the primary care literature.

Common global challenges related to transition to primary care include a lack of undergraduate preparation, limited opportunities for continuing post-licensure education, and the uniqueness of the primary care practice setting [31,32]. Professional identity and role clarity have been found to be vital to successful transition with on-going supports required to allow nurses to adjust to the shifts in scope of practice expectations associated with primary care [33]. With undergraduate programs largely focused on preparing nursing graduates for practice in acute care, initiatives to prepare and support nurses working in primary care are required [34].

Organizations are challenged to ensure essential resources are in place for newly hired nurses. Typical supports include a general orientation and opportunities for preceptorship or mentorship. Orientation is typically defined as structured time spent becoming familiar with the administrative processes of an organization or unit, and may include shadowing shifts alongside another nurse [35]. Preceptorship is a time-bound, one-way learning relationship between an experienced professional and less experienced person, often a student or new hire, in which the experienced professional shares knowledge or skills with the learner [36–38]. In contrast, mentorship is a mutually beneficial peer relationship involving socialization, bidirectional learning, and career support [39–41]. In primary care, structured mentorship helps nurses transition into practice, while enhancing autonomy, care delivery, and team collaboration [42]. It also supports newly placed RNs in adapting to the unique demands and broad skillset required in this setting [43].

A recent literature review of international evidence pertaining to the benefits of structured preceptorship programs in primary care highlights the lack of quality evidence available on this topic, further underscoring the need to undertake this study [44]. Identifying opportunities to strengthen practice supports and clarify the contributions and overall value of the nursing workforce within Family Care Teams will serve as a springboard for increasing access to care, improving patient experiences and outcomes with primary care in NL, and help catalyze initiatives across the country focused on supporting nurses’ transition into primary care settings.

### Aim

This study explores the experiences of nurses transitioning into Family Care Teams in NL by examining the resources and supports used to prepare and integrate them into teams, and by identifying the barriers and facilitators that influenced this process.

## Materials and methods

### Design

This paper is based on a qualitative descriptive study involving semi-structured qualitative interviews with NPs, RNs, and LPNs practicing in Family Care Teams in NL. We used the Consolidated Criteria for Reporting Qualitative Research (COREQ) checklist to report our work [45].

### Sampling and recruitment

We used maximum variation sampling [46] to recruit across a wide range of characteristics, including provincial health region, community size, number of years in practice, and regulatory designation. Nurses were eligible to participate if they were licensed to practice as an NP, RN, or LPN and were employed in a newly established Family Care Team setting in NL. We excluded nursing students who were undertaking a clinical placement/preceptorship in this setting.

We used a variety of recruitment methods to identify eligible participants. Study information was shared via email, newsletters, and social media by the two nursing regulatory bodies in the province, namely the College of Registered Nurses of Newfoundland and Labrador (CRNNL) and College of Licensed Practical Nurses of Newfoundland and Labrador (CLPNNL). The Canadian Family Practice Nurses Association and the Memorial University Faculty of Nursing also shared study information on their social media platforms. Lastly, we used purposive snowball sampling, whereby we asked participants to refer us to other eligible colleagues and reached out to potential participants through contacts in our professional networks. Recruitment continued until we reached data saturation [46]; that is, until interviews no longer revealed novel insights. We determined data saturation through discussions amongst the research team and the research assistant who conducted the interviews, as well as through the tracking of participant demographic characteristics. An honorarium ($50 gift card) was provided to participants upon completion of an interview.

### Data collection

We used a semi-structured interview guide (see S1 Appendix) adapted from a similar study [47], with modifications informed by research focused on nurses in primary care in NL [39,48] and transition-to-practice of novice nurses [49], as well as input from the research team and knowledge user partners. During the interviews, we asked nurses about their experience working within the Family Care Team (e.g., length of time in position, any training/education received prior to beginning position and since starting position), experience with practice supports (e.g., orientation, informal or formal mentorship, access to on-going supports such as online learning, contact lists, and members of the leadership team), current roles/activities in the Family Care Team, and barriers/facilitators to maximizing their scope of practice in this setting. We also collected demographic information such as age, gender, and type of position. The interviews were conducted by a research assistant with qualitative interviewing expertise via Zoom video conference (Zoom Video Communications Inc.). Interviews were audio recorded, transcribed verbatim, and checked for accuracy (DR, MS).

### Data analysis

We used a qualitative descriptive approach informed by qualitative content analysis and constant comparison to analyze the data [49,50]. This approach was selected to provide a rich description of nurses’ experiences transitioning into their roles within Family Care Teams while remaining grounded in participants’ accounts. Analysis involved identifying recurring patterns in participants’ descriptions and grouping similar experiences into meaningful categories [46]. To support this process, we developed a comprehensive coding template. Two team members (DR, MS) independently coded a subset of interviews reflective of different nurse designations and regions using an inductive approach. Both team members generated an initial set of codes derived directly from the data. The team members then met to compare their coding, discuss interpretations, and refine code definitions. Through an iterative process of constant comparison, similar codes were grouped into broader categories that reflected shared aspects of nurses’ transition experiences. Throughout this phase, the template was periodically updated to refine or clarify definitions, incorporate new categories as needed, and ensure consistency in application. Any discrepancies were resolved through discussion among the team. This process continued until the coding structure adequately captured the range and variation in participants’ experiences [49,50]. The final coding template was then applied to the remaining interviews using NVIVO 14 software (QSR International) to assist in the organization and management of the data. For the current paper, we focused on categories related to orientation, mentorship and practice supports provided to nurses when starting their roles in Family Care Teams, factors impacting nurses’ orientation, nurses’ perceptions around the orientation they received, and their recommendations for future transition supports.

### Rigour and positionality

We employed rigorous methods throughout the study [46,49,50], such as pre-testing interview questions and using a skilled research assistant with qualitative research expertise to conduct interviews. We completed an audit trail in order to keep meticulous documentation of all procedures and decisions made throughout the process, including the development and refinement of codes and analytic categories. We kept detailed records of field notes, drafts of the coding template, and coding disagreements and their resolutions. Throughout the interviews, we conducted member checking [51] by encouraging participant self-reflection and clarifying responses to validate meaning and ensure viewpoints were accurately represented. We captured variations in perspectives, including negative cases, through purposeful sampling to ensure rich descriptions. Our findings are presented using thick description and supportive illustrative quotes collected from the interviews [46,50,52].

Our research team was comprised of individuals with expertise in qualitative methods and health workforce research, and represented different areas of practice including nursing, family medicine, and health policy and regulation. This allowed us to leverage diverse areas of expertise and ensure that our findings are meaningful and reflective of different perspectives and experiences. All team members were involved in the development of the interview guide, ensuring that questions elicited information that were useful and relevant to support the advancement of nursing and team-based models of care in NL.

### Ethical considerations

We received ethics approval from the NL Health Research Ethics Board (HREB) (File: 20241400). Prior to starting each interview, a research assistant provided participants with a letter of information about the study and a consent form, and obtained informed consent. Informed consent was obtained verbally due to the virtual nature of the interviews, and this method was approved by the NL HREB. Verbal consent was documented within the audio recording of the interview and included within the transcription. To protect confidentiality, we assigned each participant a unique study identification code and obscured all identifying information.

## Results

A total of 25 nurses completed interviews between September and December, 2024. Interview times ranged from 28 to 68 minutes (mean = 45 minutes). Participants consisted of 6 NPs, 13 RNs, and 6 LPNs and represented all five health care zones in NL (Table 1).

**Table 1:** Participant demographics.

Participant Characteristics	N = 25 n (%)
**Nurse designation, n (%)**	
NP	6 (24.0)
RN	13 (52.0)
LPN	6 (24.0)
**Health Authority Region, n (%)** ^**a**^	
Eastern Urban	9 (36.0)
Eastern Rural	9 (36.0)
Central	3 (12.0)
Western	3 (12.0)
Labrador-Grenfell	1 (4.0)
**Gender, n (%)** ^**b**^	
Woman	24 (96.0)
Other	1 (4.0)
**Community size** ^**c**^	
Urban	8 (32.0)
Small Urban	1 (4.0)
Rural	16 (64.0)
**Highest level of education in nursing, n (%)**	
Master’s Degree (NP)	5 (20.0)
NP Diploma	3 (12.0)
Bachelor’s Degree in Nursing	11 (44.0)
RN Diploma	1 (4.0)
LPN Diploma	5 (20.0)
**Current employment status, n (%)**	
Full-time	21 (84.0)
Casual	4 (16.0)
**Specialized role in clinic, n (%)** ^**d**^	
No	17 (68.0)
Yes	8 (32.0)
**Age**	
25–34	6 (24.0)
35–44	9 (36.0)
45–54	7 (28.0)
55+	3 (12.0)
**Year of graduation** ^**e**^	
1990–1999	6 (24.0)
2000–2009	7 (28.0)
2010–2019	6 (24.0)
2020–2024	6 (24.0)
**Length of employment in primary care**	
0– < 6 months	3 (12.0)
6 months– < 1 year	2 (8.0)
1 year– < 2 years	8 (32.0)
3 years+	6 (24.0)
**Length of employment in a Family Care Team setting**	
0– < 6 months	5 (20.0)
6 months– < 1 year	4 (16.0)
1 year– < 2 years	10 (40.0)
2 years– < 3 years	4 (16.0)
3 years+	2 (8.0)

Abbreviations: NP – Nurse Practitioner; RN – Registered Nurse; LPN – Licensed Practical Nurse.

^a^The majority (63%) of the NL population resides in the Eastern Urban and Eastern Rural areas of the province [53], which is reflected in our sample; ^b^Gender was asked as an open-ended question. Other refers to any gender other than Woman and is reported this way due to low sample size; ^c^Rural < 10,000 population, Small Urban = 10,000–99,999 population, Urban > 100,0000 population [54]; ^d^Specialized roles consisted of patient care coordinators, managerial/leadership roles, diabetes nurse educators, etc.; ^e^From highest nursing degree.

### Characteristics of participants

Participants of this study described their transition to Family Care Teams in two overarching stages: 1) orientation and 2) supportive learning relationships.

### Orientation

#### Length and delivery of orientation.

The length of orientation varied for nurses across designations, with some study participants indicating they did not receive an orientation while others described completing a two- to four-week orientation prior to working with clients in the clinic setting: “*So, I actually didn’t do any orientation …at the Family Care Team once I got hired because I guess they just assumed that I* *had the prior experience*” (FPT11NP), and “*So, when we started, we actually had a two-week orientation period before we took any patients in*” (FPT18LPN)*.*

Participants noted that orientation was delivered mainly online and completed independently in an asynchronous format. Some synchronous online and in-person sessions were organized for nurses hired to work in new Family Care Teams that were not yet open. In these cases, the first week or two often involved completing modules or listening to presenters. One RN described the self-directed nature of their orientation: “*Initially, when I took the job…my first couple of weeks of work consisted of online modules and that was self-paced, self-taught. I showed up here at my building, there was …no direct manager or anything…*” (FPT08RN).

An NP recognized that their orientation likely differed from the norm as it was more structured and held in-person:

*This [orientation] might have been a little bit more organized [than previous orientations] where we had … kind of a schedule where we had presentations by certain speakers like heads of the primary care program, the Family Care Teams, and what the vision was, the mission was, things like that. So, [the current orientation to the Family Care Team] was a little bit more structured in that regard. (FPT20NP)*

An LPN recalled completing their orientation with others in a boardroom listening to guest speakers on a variety of topics such as practicing in primary care, ethics, and team dynamics. These sessions were attended by all health care professionals and administrative staff hired to work in the Family Care Teams. This participant described:

*I believe the first week was all conference style. It was in our boardroom, we had various presenters from different aspects of primary care, from ethics to… how all the disciplines interact, from social work to pharmacy to our NPs…right on down the line to our clerical. (FPT18 LPN)*

#### Content of orientation.

Many participants in this study indicated that their orientation focused largely on familiarizing them with the technology and platforms used within their Family Care Team. One NP participant, who already had some familiarity with the system from previous experience, stated:

*I had a full day, eight-hour orientation to the charting system, which I didn’t get as a student …. So, I did, after about a month, get that full orientation on how to properly chart using the system. Again, I was familiar with it, but it definitely showed some tips and tricks on how to use it. (FPT06NP)*

While some participants found value in the orientation despite having prior experience with the electronic medical record (EMR) system, others described struggling due to a lack of familiarity and inadequate time for practice. It was noted that having more opportunity to practice using the system during orientation would have been of benefit to new hires. An LPN participant who had undergone this training a year prior shared:

*EMR, our charting system, we need better orientation for that. Five hours in front of a screen in a group of ten people being led through at record speed because they didn’t allow enough time, didn’t give us the proper training.…. we’re a year in, and it’s only now that we’re really getting comfortable with the EMR. (FPT18LPN)*

Beyond technological training, several participants described orientation sessions that focused on the broader structure and content of care delivery within Family Care Teams. For example, participants described content based on the Family Care Team and associated framework, disease processes, assessments, and provincial resources or those specific to their region or community:

*I did do some courses on the framework of the Family Care Team and that kind of stuff. But as for …the disease prevention/promotion, I haven’t done anything like that. I did do the [specific chronic disease prevention and screening program], which helps me look at… provincial screening…I did do some self-management and chronic disease workshops, but other than that, the orientation was fend for yourself. (FPT27 RN)*

In addition to being introduced to available resources, another RN noted that their orientation included training on referral processes and workshops related to approaches around common conditions they would see in clients attending the Family Care Team. This participant expressed: “*We did have specific training in…hypertension, a little bit diabetes, [and] we had some resources on cancer… but basically it was an introduction to the programs and services*” *(FPT08RN).* Several participants spoke about being oriented to resources that were discontinued not long after transitioning to their role. One LPN stated: “*I was actually trained in what’s called [specific chronic disease prevention and screening program]v. Unfortunately, now that is done…the funding for it was cut… so we were told now that we can’t even use [it]*” (FPT18LPN).

### Supportive learning relationships

Many participants reported engaging in different forms of supportive relationships during their orientation and described them as either mentorship, preceptorship, or shadow shifts with colleagues. While mentorship and preceptorship represent separate and distinct forms of support, study participants often used the terms interchangeably or used the term ‘mentorship’ to describe shadow shifts or extended preceptorship experiences. The amount of time spent in these activities differed across nurse designations and by Family Care Team clinic, with one NP indicating that their mentorship lasted “*just a few days*” *(FPT28NP)*, an RN stating “*3 or 4 days*” *(FPT30RN)* and an LPN recalling that “*there was nothing... formal with a preceptor*” *(FPT18LPN)*. Participants described not always being paired with a person with the same nursing designation or within the same professional group. Some pairings were consistently with the same person while others had shadow shifts with several different people. Participants shared varied experiences with learning relationships, with some reporting structured and formal pairings, while others characterized their experiences as informal and occurring on an ad-hoc basis. One participant described their experiences with this approach:

*You have to review the current policies for the workplace, and then you do get paired with a mentor and then you can shadow for a couple of days until you’re comfortable. And then you can kind of go on your own with the mentor on site to ask questions. (FPT28NP)*

A new graduate participant who had previously completed a student placement in the same Family Care Team reported a different mentorship experience than participants who were entirely new to the setting, as it was assumed that their familiarity with the clinic environment reduced their need for additional support. The new graduate NP reflected on their perception of a brief transition experience:

*Once I had been hired, I had a few days’ orientation just shadowing one of the nurse practitioners there at the clinic. But because I had so much clinical time as a student, I didn’t get weeks’ long orientation because I felt pretty comfortable with the process. (FPT06NP)*

In contrast, some participants did not have the opportunity to be paired with a more experienced primary care nurse during their transition, which was felt to contribute to role ambiguity. For example, one participant reflected on the lack of structured support and role clarity: “*...we never… had a chance to job shadow. I was never sent to another established clinic to see what the other LPNs were doing. It was, ‘here you go, you’re in, do it’.... there was no clear roles*” *(FPT18LPN).*

Another participant stated that, although they were paired with another RN for a few days, there were limitations as to how much they could learn as their peer had less than a year of experience in their role:

*After that 3 or 4 days, I was put with another RN who had been working in the clinic for the last six, eight months and then I kind of job shadowed with her doing intakes and virtual care clinic and profiling, she showed me how to do the profiling of the patients. But that was basically it. (FPT30RN)*

In some cases, others chose not to engage in the mentorship that was offered as this was not perceived as helpful given that it would involve shadowing someone in a role that differed from their own, or because the assigned mentor was also new to their position and also lacked role clarity. A participant shared that, although they were offered a mentorship opportunity, they felt it was not helpful due to the experience level of the available mentor:

*So, with regards to the orientation, I spent one day, I went to another Family Care Team and spent one day with another chronic disease nurse who had just started as well and didn’t really know her role. So, my manager would let me go back up there with her, but I didn’t see the sense of it because she was, she didn’t know, she was still in the same boat I was. (FPT03RN)*

Some participants expressed that receiving mentorship was either not possible or not particularly useful due to being hired to a Family Care Team that was newly opened. An RN described how the absence of colleagues in similar roles meant that there was no one available to provide guidance or role modeling:

*I didn’t get a formal mentorship because there was nobody in my position prior to coming to this [Family Care Team]. Because our program is brand new, brand new for our area, brand new for our province, myself and everybody else on the team didn’t have anybody to lead us. (FPT16RN)*

While some participants were unable to access effective mentorship due to the novelty of their Family Care Team, others expressed a desire to receive mentorship at a different clinic outside of their team that was more established. One RN stated: “*I do think it would have been beneficial if they … brought me to a different area of Newfoundland where the Family Care Team is established to … understand that role there*” *(FPT05RN).*

Although some nurses reported a lack of mentorship, this was not always expressed as problematic. In some areas, NPs entering into these roles brought prior primary care experience, which appeared to mitigate the need for mentorship during their transition into the Family Care Team. These experienced nurses were referred to as legacy nurses, with one NP noting: “*… in our area, we were fortunate that we already had legacy nurse practitioners*” *(FPT26 NP).*

Participants expressed that there could be differences in mentorship needs based on nurse designation. Specifically, NPs were noted to have a more comprehensive mentorship. One participant stated: “*I think having those hour-long appointments with ensuring that there is a* *senior NP or a physician available... [you] felt really well-supported,... they made the transition much better*” *(FPT12NP).*

## Discussion

Our study explored the experiences of nurses transitioning into Family Care Teams, a team-based model of primary care across NL. Results indicated variability in the structure and delivery of orientation, limited opportunity for mentorship, and a lack of clarity in nurses’ roles in Family Care Teams. Variability in participant demographics was also noted and reflects the need to consider a common transition framework that can be adapted to each new hire’s specific learning needs. This approach reflects recently designed frameworks to support the transition of newly hired nurses in acute and intensive care settings [55,56]. Furthermore, resources available to all Family Care Teams in NL are required to better facilitate the successful integration and contribution of NPs, RNs, LPNs, as these team-based practices continue to expand in the province. While team-based primary care is relatively new in NL, similar models have been well-established in other provinces [57–59]. Insights from these previously established models may inform ways to build trust and enhance role clarity within teams, including strategies for effective communication, clearly defined responsibilities, and collaborative decision-making to support nurses in contributing fully to patient care and team functioning. Results of this study also highlight a notable gap in the preparation of nurses to take on roles in Family Care Teams, suggesting the need for a national, overarching framework for nurses who transition to team-based primary care settings.

The implementation of team-based primary care, such as Family Care Teams in NL, represents a shift from the traditional physician-led clinics to an interprofessional model of primary care [60]. This model is known to improve access, quality of care, and patient outcomes [3]. However, our findings show that nurses transitioning to Family Care Teams face significant gaps in preparation and support, which may compromise the achievement of these desired outcomes such as access to primary care services, continuity of care, improved health outcomes, patient satisfaction, and lower costs [3,7]. The brief, inconsistently structured orientation processes described by nurses participating in our study were largely focused on technology and delivered asynchronously, which differs from reported onboarding practices in acute care settings that focused more on the clinical learning environment and supervision [61]. However, the remote aspect described by many participants in this study may reflect the shift to online orientation that occurred during the recent pandemic [62]. Approaches used during this shift should be considered and adapted to the geographical setting of the Family Care Teams [63]. The approach to orientation described in this study did not adequately prepare newly hired nurses to take on their role within the Family Care Team, with many taking up to year to build confidence in organizational technology and processes such as the electronic health records system. Methods used to orient nurses to these types of systems, as well as clinical aspects of their roles in other care settings, have been published in Canada [62] and globally [61,64], and could be applied to Family Care Teams in NL. Participants in this study also frequently reported uncertainty and confusion around their roles and responsibilities within the care team. This echoes results from similar studies focused on the underutilization of nurses in primary care [32,65]. The importance of preparing student nurses to take on the increasing number of positions in primary care in NL is paramount. Experiences shared by a new graduate nurse in our study indicates that their transition-to-practice could be facilitated through the completion of student placements in these settings to allow for greater familiarity and orientation to the role. Increasing these clinical opportunities for student nurses and introducing theoretical and conceptual aspects of primary care in undergraduate nursing programs would assist in preparing nurses to successfully take on these roles, facilitate recruitment into the increasing number of positions on these teams, and help stabilize the nursing workforce in primary care [5].

Participants in this study identified mentorship and peer support as facilitators when available and as missed opportunities when absent. While some nurses had access to supportive learning relationships, such as shadow shifts or contact with a legacy nurse, most did not. Many nurses were hired into newly established teams where no experienced primary care nurses were available to assist with orientation or mentorship. This lack of professional expertise and role modeling led to self-doubt in some new hires and contributed to role ambiguity, particularly among RNs and LPNs. These findings reflect identified challenges in transitioning to primary care settings. Studies have indicated that while clinical independence and a broad knowledge base are expected of new hires, these can be difficult to achieve due to the prevailing focus on acute care in current nursing curricula [66–69]. A transition framework that mirrors practices observed in acute care across the country and internationally that is consistently applied in all Family Care Teams in NL may help to address many of the challenges identified by participants in our study [70]. As these nurses suggested, pairing new hires with experienced nurses with the same designation in established Family Care Teams could increase role clarity and build clinical competence. These supports are especially important in the context of NL and other jurisdictions where team-based primary care models are still emerging and mentorship capacity is limited.

In NL and across the country, there remains a lack of primary care-focused content in undergraduate curricula and limited post-licensure training specific to the primary care setting [71,72]. A recent environmental scan identified various programs that exist; however, most were region/province-specific or focused on a singular practice activity or competency domain [12]. To address this gap between formalized training and the professional practice expectations of nurses in primary care, the Team Primary Care Nurse (TPCN) Post-Licensure Educational Program was recently launched as a context-specific solution [73]. Embedding this program in Family Care Teams’ onboarding processes for newly hired nurses may assist to standardize practices, increase foundational knowledge, and improve role clarity. Given that mentorship capacity within Family Care Teams is currently limited, utilizing existing evidence-based resources such as the TPCN Post-Licensure Educational Program could act as an effective option to ensure consistency and quality of care in primary care settings as these team-based models become increasingly established in NL.

### Limitations

This study examined the experiences of nurses transitioning into Family Care Teams in a single Canadian province. As such, findings may not be generalizable to other Canadian provinces or to international contexts, where the structure, scope, and implementation of primary care teams may differ. In NL, Family Care Teams represent a relatively new model of care that is still evolving, with implementation occurring at different rates across regions. As these teams become more established and standardized, nurses’ orientation experiences may change; therefore, the findings reflect a snapshot of early implementation and may not capture longer-term developments or changes to the orientation process. A strength of this study is the inclusion of LPN perspectives, as this is a noted gap in existing literature. As with all qualitative interviews that consist of self-reported data, the findings from this study may be subject to recall bias [74] and social desirability bias [75]. This was mitigated through the avoidance of leading questions, the application of consistent prompts, and the use of an experienced interviewer that encouraged participants to reflect on their experiences and share candid opinions.

### Implications

Results of this study indicate further work is needed to develop a consistent orientation and mentorship program for nurses transitioning to team-based primary care. In NL and other provinces where primary care teams are becoming more established, the ability to mentor within these provinces will increase. Until then, including the TPCN Post-Licensure Educational Program as part of a standardized training approach in Canada to support and facilitate the transition of nurses into these much-needed teams is recommended [76]. To complement the TPCN educational program, it is suggested that government and health decision makers invest in developing a formal structure for primary care nursing orientation and mentorship that can be applied across provinces and jurisdictions, based on individual needs and context. Furthermore, increasing the number of student placements in primary care will expose and orient future nurses to this practice setting. Additional research is needed to explore how orientation to new roles affects nurses’ role clarity and their ability to contribute effectively to team-based models of primary care.

## Conclusions

This study provides valuable insights into the experiences of NPs, RNs, and LPNs who transitioned into Family Care Teams in NL. Our findings highlight gaps in orientation and mentorship for nurses, representing opportunities to better support nurses transitioning to these roles in the expanding number of Family Care Teams across the province. Recommendations include integrating primary care content into undergraduate nursing programs, ensuring all newly hired nurses complete the TPCN National Educational Program, and implementing a tailored approach to transition for primary care nurses that emphasizes role clarity among interprofessional team members. Future research should prioritize the implementation and evaluation of these practice recommendations, while also exploring ways to adapt transition supports that can be scaled and adapted to different regional, national, and international primary care contexts.

## Supporting information

S1 Appendix Interview guide. (DOCX)
