# Supplementary material for: ‘Fend for yourself’: Nurses’ experiences transitioning to Family Care Team practice settings in Newfoundland and Labrador
Source: PLoS One. 2026 Mar 24;21(3):e0345818. doi: 10.1371/journal.pone.0345818 (PMC13012503; doi:10.1371/journal.pone.0345818)
Supplement: S1 Appendix — (DOCX) [file pone.0345818.s001.docx]

**S1 Appendix. Interview Guide**

1. First, I would like to collect some basic demographic information:

- Length of employment in this Family Care Team

- Position type currently held: FT □ PT □ Casual □ / Permanent □ Temporary □

- Length of time employed in primary care overall

- Describe your previous nursing experience?

- Age

- Gender

- Year of graduation

- Highest level of education: Undergraduate □ Masters □ PhD □

- Highest level of education in nursing: Undergraduate □ Masters □ PhD □

2. Can you tell me about your current role in this primary care practice?

- Obtain a brief overview of current role

- Type of population served

- Focus on prevention, health promotion, and population health

- Who are other members of the primary care team? (e.g., number of other providers and roles)

- Referrals to other team members/healthcare providers

- Degree/extent of collaboration between other healthcare providers

3. Could you please describe your transition to primary care and the context around how and why you came to accept the position?

- Context around when you first arrived at your position

-Your motivation/vision at the time of accepting the position

The following two questions ask about practice supports and resources, both related to when you first started/transitioned to role and beyond.

4. What type of preparation or support did you receive to work in your current role within this Family Care Team during your transition to practice?

- Orientation (How were you oriented? With whom? How long? Was there a theoretical component?)

- Continuing education

- Informal/formal mentorship

- Other

- If you worked in acute care or other practice setting prior to primary care, please describe any differences in your transition experience (orientation, buddy shifts, mentorship)

- Did your experience meet your transition/learning requirements?

5. Since starting your position, what type of practice supports have you received to work in your role within this Family Care Team?

- Toolkits, guides, resources

- Continuing education

- Informal/formal mentorship

- Other

6. How has your nursing education (basic and continuing education) prepared you for this role and provided you with the ability to work to the full extent of your knowledge and skills in a Family Care Team? What gaps do you see in your nursing education for working in this setting?

- Appropriateness of education

- Gaps in knowledge/skills

- Learning needs

- Preparation to work in collaborative practice with other healthcare providers

- Gaps between what you were taught and how you practice

7. Previous research focused on nursing practice within collaborative primary care teams has found considerable role ambiguity, in that nursing roles were not particularly clear. As a result, nurses who work in primary care settings were not necessarily using the knowledge and skills from their basic education as effectively as they could be.

- How is this similar or different to information you have read/heard?

- How is this similar or different to the experience you have had in primary care?

8. How is your current position within the Family Care Team funded?

- Salary/hourly rate?
 - Do you know where the funds come from to cover your salary/hourly pay?

9. What do you perceive as barriers to optimizing your contribution to patient care within this setting? What do you perceive as facilitators to optimizing your contribution to patient care within this setting?

10. In an ideal world, what would transition to primary care practice look like for nurses?

- What supportive resources would be available?

- Who would provide them?

11. Do you have any additional information that you would like to share?
